# Genetic diversity and structure of Chinese grass shrimp, *Palaemonetes sinensis*, inferred from transcriptome-derived microsatellite markers

**DOI:** 10.1186/s12863-019-0779-z

**Published:** 2019-10-11

**Authors:** Yingying Zhao, Xiaochen Zhu, Zhi Li, Weibin Xu, Jing Dong, Hua Wei, Yingdong Li, Xiaodong Li

**Affiliations:** 1 Key Laboratory of Zoonosis of Liaoning Province, College of Animal Science and Veterinary Medicine, Shenyang Agricultural University, Shenyang, 110866 China; 2 College of Aqua-life Science and Technology, Shanghai Ocean University, Shanghai, 200090 China; 3 Panjin Guanghe Crab Industry Co.Ltd., Panjin, 124000 China

**Keywords:** *Palaemonetes sinensis*, Genetic diversity, Genetic structure, Transcriptome, Microsatellite

## Abstract

**Background:**

The Chinese grass shrimp, *Palaemonetes sinensis*, is an economically important freshwater shrimp in China, and the study of genetic diversity and structure can positively contribute to the exploration of germplasm resources and assist in the understanding of *P. sinensis* aquaculture. Microsatellite markers are widely used in research of genetic backgrounds since it is considered an important molecular marker for the analyses of genetic diversity and structure. Hence, the aim of this study was to evaluate the genetic diversity and structure of wild *P. sinensis* populations in China using the polymorphic microsatellite makers from the transcriptome.

**Results:**

Sixteen polymorphic microsatellite markers were developed for *P. sinensis* from transcriptome, and analyzed for differences in genetic diversity and structure in multiple wild *P. sinensis* populations in China. Totally of 319 individual shrimps from seven different populations were genotyped to find that allelic polymorphisms varied in two to thirteen alleles seen in the entire loci. Compared to other populations analyzed, the two populations including LD and SJ showed lower genetic diversity. Both the genetic distance (*D*) and Wrights fixation index (*F*_*ST*_) comparing any two populations also indicated that LD and SJ populations differed from the other five populations. An UPGMA tree analysis showed three main clusters containing SJ, LD and other populations which were also confirmed using STRUCTURE analysis.

**Conclusion:**

This is the first study where polymorphic microsatellite markers from the transcriptome were used to analyze genetic diversity and structures of different wild *P. sinensis* populations. All the polymorphic microsatellite makers are believed useful for evaluating the extent of the genetic diversity and population structure of *P. sinensis*. Compared to the other five populations, the LD and SJ populations exhibited lower genetic diversity, and the genetic structure was differed from the other five populations. Therefore, they needed to be protected against further declines in genetic diversity. The other five populations, LP, LA, LSL, LSY and LSH, are all belonging to Liaohe River Drainage with a relatively high genetic diversity, and hence can be considered as hot spots for in-situ conservation of *P. sinensis* as well as sources of desirable alleles for breeding values.

## Background

*Palaemonetes sinensis* (Sollaud, 1911), also known as Chinese grass shrimp, a small freshwater shrimp belonging to the Palaemonidae family distributed in China, Myanmar, Vietnam, Japan, southeastern Siberia and Sakhalin and has both ecological and ornamental value [1–3]. In China, *P. sinensis* is commonly distributed in the Liaoning, Jilin, Heilongjiang, Hebei, Jiangsu, Fujian and Yunnan provinces [1]. *P. sinensis* is an attractive shrimp due to its striking appearance, appealing flavor [4] and use as bait for sport fishing [3]. Due to its delicious meat and high nutritional value, *P. sinensis* is very popular in both domestic and foreign markets [3–6]. However, as a consequence of environmental pollution and overharvesting, the *P. sinensis* population has gradually diminished. As such, there has been interest in *P. sinensis* aquaculture to potentially alleviate fishing pressure on the wild population by meeting consumer demand with farmed shrimp and through stock enhancement. Nevertheless, there are only a handful of studies investigating *P. sinensis* morphology and more work is needed to understand the biology of this species [3, 6]. At present, studies on the *P. sinensis* using molecular biology are rarely reported, only some studies have used microsatellite markers in other species of the Palaemonidae family, including *Macrobrachium rosenbergii* [7] and *Macrobrachium nipponense* [8]. In the past decade, microsatellite markers have been widely used in the genetic background research [9], and have been considered as very important molecular genetic markers for the analysis of genetic diversity, genetic structure and construction of genetic linkage maps. With the development of sequencing technology, vast quantities of transcriptome information were obtained, including of the identification of microsatellite markers. Ma et al. [10] identified 129 polymorphic microsatellite markers from transcriptomic analysis and analyzed their relationship to growth performance in Mud Crab (*Scylla paramamosain*). Research in related species of snapping shrimps with highly duplicated genomes proved that microsatellites acquired from the transcriptome were more likely to work effectively than those developed through traditional methods from the genome [11]. However, compared to other aspects of massive transcriptome data, the roles of transcriptome-derived microsatellite markers have not been investigated thoroughly. In prior work, a total of 17,019 microsatellite markers were obtained from the transcriptome of the *P. sinensis* [4]. Moreover, there are no studies investigating molecular marker based genetic diversity of *P. sinensis*, which has the potential to aid in the conservation and improvement of this shrimp species. In view of the broad distribution of its wild populations, the questions over whether they are genetically similar or do exhibit differences attributable to their locations or drainage basins are still unsolved. The objectives of the current study were to validate variations of transcriptome-derived microsatellite markers and to analyze their underlying genetic background of different wild *P. sinensis* populations. It is hoped that this work could make a positive contribution to the molecular genetic analyses of *P. sinensis* populations and eventually serve as a basis for the improvement and sustainable conservation of *P. sinensis* aquaculture in China.

## Results

### Polymorphisms of microsatellite loci

**Fig. 1:**
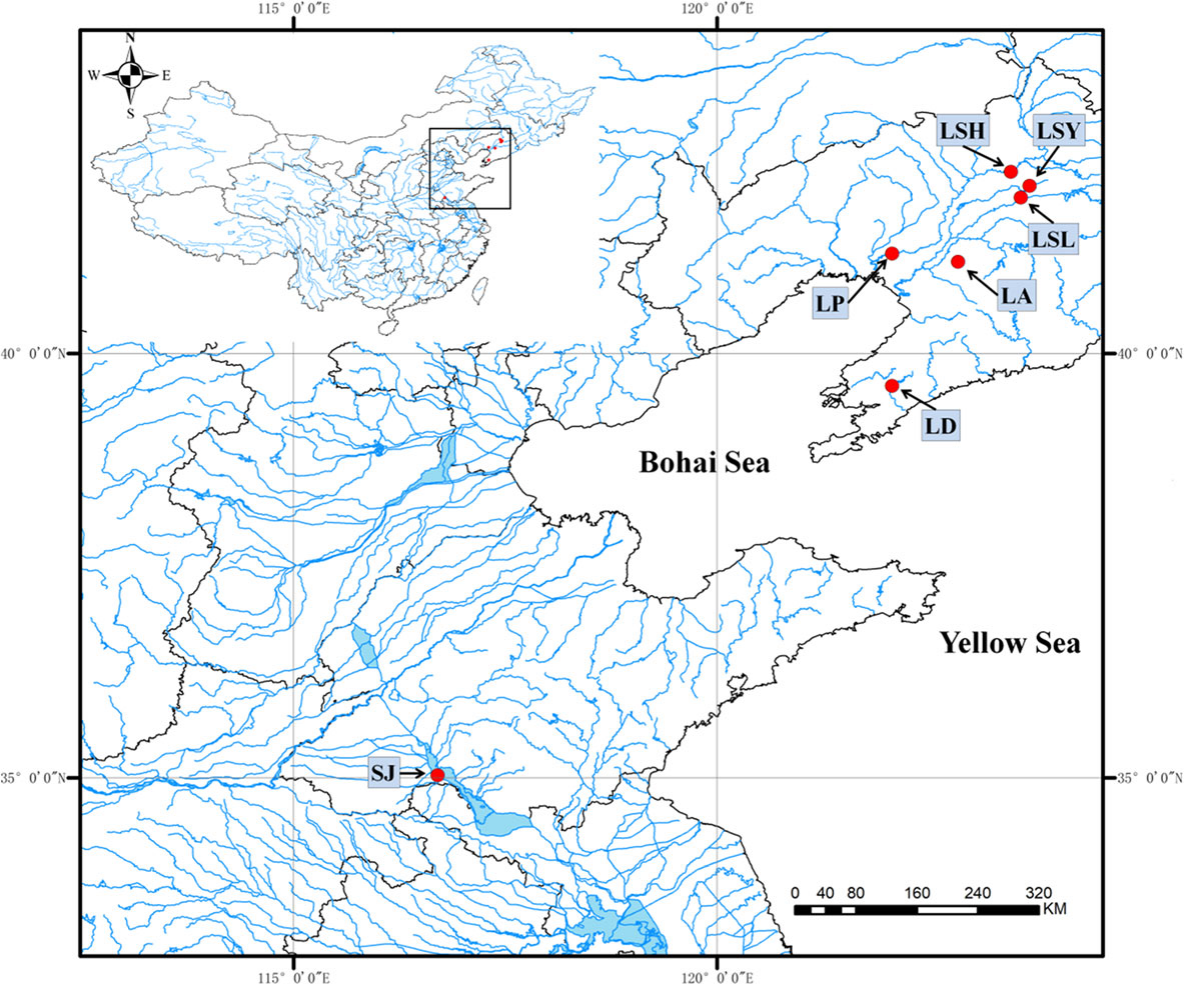
Map of *P. sinensis* samples collection sites. (see Table 1 for full description of the populations)

Seven wild populations of *P. sinensis*, totaling 319 individual shrimps (Fig. 1, Table 1), were screened for 16 microsatellite loci which were polymorphic in all populations using the 0.95 allele frequency criterion. The characteristics of the 16 microsatellite loci were summarized in Table 2. The most polymorphic locus was c1747_g1_i1 among the 16 loci, with the highest *N*_*A*_ (13), *N*_*E*_ (3.628), *H*_*e*_ (0.724) and *A*_*r*_ (7.478), and c2591_g1_i1 was the least polymorphic locus, with the lowest *N*_*A*_ (2), *N*_*E*_ (1.006), *H*_*e*_ (0.006) and *A*_*r*_ (1.213) values.

**Table 1:** List of populations used in this study and their geographic position of *P. sinensis*

Population	*No.*	Location	Geographic position	
LD	48	Liaoning Dalian Sha River	39°37′19.7″N	122°04′03.0″E
LP	43	Liaoning Panjin Shuangtaizi River	41°10′49.6″N	122°04′01.4″E
LA	48	Liaoning Anshan Yangliu River	41°04′57.8″N	122°50′51.7″E
LSL	48	Liaoning Shenyang Longwei Lake	41°50′33.7″N	123°35′22.3″E
LSY	48	Liaoning Shenyang Yangshi reservoir	41°58′40.8″N	123°41′27.5″E
LSH	48	Liaoning Shenyang Huangjia Liao River	42°08′46.6″N	123°28′21.0″E
SJ	36	Shandong Jining Dushan Lake	35°01′59.5″N	116°42′09.2″E

*No.*: sample number of each population

**Table 2:** Characteristics of the microsatellite markers and their genetic variation statistics in *P. sinensis*

Locus name	Forward primer sequence(5′-3′)	Reverse primer sequence(5′-3′)	Repeat unit	Annealing temperature	Size range (bp)	*N*_*A*_	*N*_*E*_	*H*_*o*_	*H*_*e*_	*A*_*r*_	*r*	*F*_*IS*_	*P*_*HWE*_
c119_g1_i1	F:GGGAGGAGTTTCGTATTCGTT	R:TTTTGCCTCCTCTTTTCAGG	(TGA)_5_	54 °C	251–263	4	1.096	0.072	0.087	2.462	0.021	0.160	0.278
c251_g1_i1	F:TGAATGACGTCACGGTTGTT	R:CGTGGAGATGTGGGAGTGTA	(AC)_8_	54 °C	236–248	7	1.654	0.335	0.395	4.479	0.042	0.134	0.008^**^
c317_g1_i1	F:GGGATGGCCTGTAAAGTTGA	R:CACCTCCAGTCTCAAAAGCC	(CAT)_5_	54 °C	269–275	5	1.128	0.100	0.114	2.946	0.014	0.072	0.144
c341_g1_i1	F:GCTGTTGAGCACTTTCATTCA	R:GGGCTCATGTTAAATCCAAGA	(TA)_7_	54 °C	258–272	9	1.938	0.295	0.484	4.407	0.068	0.211	0.000^**^
c864_g1_i1	F:GATGCGATAGATGACGCCTT	R:ATGCTTAGGTGGTGGAGTGG	(TGA)_6_	54 °C	263–272	4	1.204	0.185	0.169	2.563	0.000	−0.178^*^	0.973
c1089_g1_i1	F:GTGATCCAGGTCGAACACAA	R:TTCTACCCGGTGCTTTTGAC	(GGA)_5_	54 °C	196–201	5	2.005	0.430	0.501	2.707	0.044	0.055^*^	0.000^**^
c870_g2_i1	F:TGGTTCTATCCGCAGATTCC	R:CCTTCCTGTTGGTGGTGTCT	(TCA)_6_	54 °C	189–192	2	1.137	0.072	0.120	1.994	0.003	0.065^*^	0.000^**^
c679_g1_i1	F:TTTGCTGGCAAGGAACATTA	R:AGATCACCCCTCTTCCTTCC	(AGG)_5_	54 °C	92–114	4	2.887	0.596	0.654	3.838	0.022	0.035^*^	0.194
c792_g1_i1	F:TTGGCTTCAGCAAATCCTTT	R:TCCTTTCGCTTTTGATACCAG	(AT)_6_	54 °C	198–208	6	1.643	0.235	0.391	2.991	0.094	0.325^*^	0.000^**^
c102_g1_i1	F:TGGTGATGGGGATTTCCTTA	R:TTCTTTTATTGTCTGATTATGCCA	(AT)_6_	54 °C	256–264	5	1.930	0.282	0.482	4.813	0.094	0.246^*^	0.000^**^
c1157_g2_i1	F:ATGTCGGAACGGACAGAAAA	R:TGAAGCAGAGGAAACGTTGA	(GA)_7_	54 °C	105–109	3	2.305	0.254	0.566	3.000	0.093	0.333^*^	0.000^**^
c1198_g1_i1	F:TCTGGAAAATTTTTGGGCAC	R:GAAATTAAGGTTTAGCACATTCTCC	(TA)_7_	54 °C	165–201	6	3.136	0.430	0.681	4.326	0.131	0.320^*^	0.000^**^
c1730_g1_i1	F:TGAAATTCCAGGAAAGGCTG	R:CGTGTCCTTCCACGAAAGAG	(GGA)_5_	54 °C	191–209	13	3.291	0.511	0.696	6.042	0.043	0.094	0.055
c1747_g1_i1	F:CAGGAGACCATGTAGAATTACGC	R:TTTGATGACAACGTGGCACT	(CA)_7_	54 °C	186–222	13	3.628	0.542	0.724	7.478	0.046	0.081^*^	0.000^**^
c2017_g1_i1	F:GTGATTTGACGTGACGAACG	R:AGACTGCAGGAGAGGCTCAG	(GAT)_6_	54 °C	253–271	5	1.139	0.129	0.122	3.021	0.000	−0.097^*^	1.000
c2591_g1_i1	F:GCATCAGAAACTTGGAGCCT	R:ACAGTAGCTAGGGGGCTTGG	(TG)_10_	54 °C	148–154	2	1.006	0.006	0.006	1.213	0.001	−0.013^*^	1.000

*N*_*A*_: number of alleles; *N*_*E*_: number of effective alleles; *H*_*o*_: observed heterozygosity; *H*_*e*_: excepted heterozygosity; *A*_*r*_: allele richness; r: null allele frequency; *F*_*IS*_: inbreeding coefficient, ^*^Significance value from 95% confidence interval; *P*_*HWE*_: *P*-value for deviation from Hardy-Weinberg equilibrium, ^**^ highly significant (*P* < 0.01)

### Hardy-Weinberg equilibrium test and linkage disequilibrium

Nine of the sixteen loci showed a highly significant departure from Hardy-Weinberg equilibrium (HWE) (*P* < 0.01), whereas the other seven loci showed no significant differences (Table 2). All populations, except for LSY, showed a highly significant deviation from HWE (*P* < 0.01) (Table 3). However, from the outcome of deviation from HWE for each locus in each population (Additional file 1: Table S1), the above extreme results are likely to be the consequence of mixing analysis for multi-populations. In over 420 pair-wise comparisons for linkage disequilibrium (Ld) among 16 loci in all *P. sinensis* populations, there were seven significant comparisons in LA and LSL, six significant comparisons in LP, LSH and SJ, as well as five significant comparisons in LSY (Table 3). In general, no consistencies were found to be significant in pair-wise comparisons for Ld indicating that there was no linkage among these loci and their inclusion will not affect the results of genetic variability [12].

**Table 3:** Summary statistics of the genetic diversity of *P. sinensis* populations

Populations	*N*_*A*_	*N*_*E*_	*H*_*o*_	*H*_*e*_	*A*_*r*_	*F*_*IS*_	*P*_*HWE*_	Ld	*N*_*e*_	M ratio
LD	3.3	1.604	0.255	0.278	3.117	0.093^*^	0.000^**^	0	224.1	0.860
LP	3.2	1.846	0.288	0.350	3.043	0.189^*^	0.000^**^.	6	Infinite	0.908
LA	3.0	1.839	0.217	0.316	2.861	0.322^*^	0.000^**^	7	106.1	0.862
LSL	3.6	1.826	0.324	0.379	3.418	0.156^*^	0.000^**^	7	1941.1	0.856
LSY	2.8	1.752	0.316	0.331	2.749	0.056	0.464	5	165.5	0.867
LSH	3.3	1.864	0.369	0.374	3.163	0.026	0.000^**^	6	Infinite	0.826
SJ	2.6	1.441	0.158	0.226	2.563	0.314^*^	0.000^**^	6	137.8	0.895

*N*_*A*_: number of alleles; *N*_*E*_: number of effective alleles; *H*_*o*_: observed heterozygosity; *H*_*e*_: excepted heterozygosity; *A*_*r*_: allele richness; *F*_*IS*_: inbreeding coefficient, ^*^Significance value from 95% confidence interval; *P*_*HWE*_: *P*-value for deviation from Hardy-Weinberg equilibrium, ^**^highly significant (*P* < 0.01); Ld: number of loci in linkage disequilibrium; *N*_*e*_: Estimates of effective population size with 95% confidence intervals; M ratio, the mean ratio of the number of alleles to the range in allele size

### Genetic diversity among populations

Data for all parameters of genetic diversity for the seven *P. sinensis* populations were shown in Table 3. The LSL population presented the highest *N*_*A*_, *H*_*e*_ and *A*_*r*_ values, while the LSH population exhibited highest *N*_*E*_ and *H*_o_ values. In the LD, LP, LA, LSL and SJ populations, *F*_*IS*_ coefficients varied from 0.093 to 0.322, suggesting significant deficiencies of heterozygotes with 95% confidence interval. In the other two populations, *F*_*IS*_ values did not show significantly different from zero.

Estimated of effective population size (*N*_*e*_) and the mean ratio of the number of alleles to the range in allele size (M ratios) of each population were also listed in Table 3. The *N*_*e*_ values for LP and LSH showed to be the highest (infinite), whereas the lowest *N*_*e*_ value was 106.1 as shown in LA. M ratios among all populations ranged from 0.826 to 0.908, which revealed that no population experienced reduction in effective size. Reduction in effective size only occurs when M < 0.68 [13]. Potential genetic bottleneck analysis performed using a Wilcoxon sign-rank test under TPM with 90% single-step mutations, showed that all populations exhibited normal L-shaped distribution and might not have experienced a bottleneck recently (*P* > 0.05).

### Genetic divergence and distance between populations

The pairwise Wrights fixation index (*F*_*ST*_) and Cavalli-Sforza and Edwards’ genetic distance (*D*) [14] values were shown in Table 4, revealing significant differences among all populations. The analysis of molecular variance (AMOVA) revealed that genetic variation within and among populations was 82.76 and 17.24%, respectively. In addition, the variation among populations was found to be significant (*P* < 0.01) (Table 5).

**Table 4:** Pairwise of *F*_*ST*_ (above diagonal) and Genetic distance (below diagonal) for *P. sinensis* populations

Populations	LD	LP	LA	LSL	LSY	LSH	SJ
LD		0.159^**^	0.190^**^	0.197^**^	0.225^**^	0.200^**^	0.312^**^
LP	0.186		0.064^**^	0.043^**^	0.096^**^	0.094^**^	0.278^**^
LA	0.167	0.119		0.081^**^	0.129^**^	0.138^**^	0.330^**^
LSL	0.211	0.139	0.133		0.036^**^	0.070^**^	0.306^**^
LSY	0.193	0.166	0.164	0.125		0.092^**^	0.360^**^
LSH	0.192	0.149	0.145	0.120	0.148		0.311^**^
SJ	0.166	0.178	0.205	0.243	0.235	0.226	

^**^Highly significant level of differentiation (*P* < 0.01)

**Table 5:** Analysis of molecular variances (AMOVAs) among seven *P. sinensis* populations

Source of variation	*d.f.*	sum of squares	variance components	percentage variation
among population	6	315.241	0.548^**^	17.24
within population	318	1660.762	2.632	82.76
total	324	1976.003	3.180	

*d.f.*: degrees of freedom; ^**^highly significant source of variation (*P* < 0.01)

Basically, the *F*_*ST*_ values among the seven populations reflected their geographic relationships. Firstly, there was a very great genetic differentiation between SJ (the only population from Huaihe Drainage Basin in Shandong Province) and the other six populations (all in Liaoning Province) (*F*_*ST*_ > 0.25). Secondly, LD (Related River Drainage Basin) and all populations from Liaohe River Drainage Basin (LP, LA, LSL, LSY and LSH) exhibited a great genetic differentiation between (0.15 < *F*_*ST*_ < 0.25). Finally, in Liaohe River Drainage Basin, there was a moderate genetic differentiation among LP, LA, LSL, LSY and LSH (0.05 < *F*_*ST*_ < 0.15), meanwhile, LSL and LP, LSL and LSY populations showed little genetic differentiation (*F*_*ST*_ < 0.05) [15] A comparison of *F*_*ST*_ values revealed significant differences among the populations (*P* < 0.01).

As analyzed by the Mantel test, the correlation between geographical distances and a pairwise comparison with genetic distances (*D* values) was significantly correlated (*r* = 0.803, *p* = 0.001). The *D* value between the populations displayed similar pattern as *F*_*ST*_ values. For example, most *D* values between SJ and the other six populations including SJ vs LA, SJ vs LSL, SJ vs LYS, and SJ vs LSH, were larger than 0.20, suggesting that they were genetically disparate populations [16]. In addition, the *D* values between LD and populations of Liaohe Drainage Basins were also higher than those between latter populations.

**Fig. 2:**
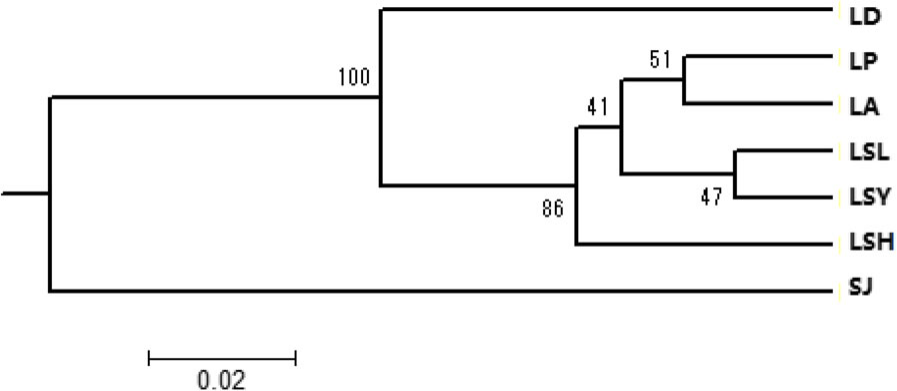
UPGMA dendrogram of genetic relationship among the seven *P. sinensis* populations using genetic distance over 1000 replicates. Numbers at the roots of the branches are bootstrap values

The UPGMA dendrogram constructed according to the *D* values was shown in Fig. 2. The seven populations formed three major clusters, LD, SJ and the other five populations. Among the five populations, LP and LA as well as LSL and LSY populations were more similar.

Gene flow between locations ranged from 0.608 (LA into LD) to 3.545 (SJ into LP). Most long-term gene flow between populations were symmetric (the 95% credible intervals overlapped in their pairwise comparisons), except those between SJ and LP, LSY and LSL, and SJ and LSY (Additional file 2: Table S2). As for cumulative gene flow, most populations received more migrants than they supplied, whereas SJ and LSL present the opposite results (Additional file 3: Table S3).

**Fig. 3:**
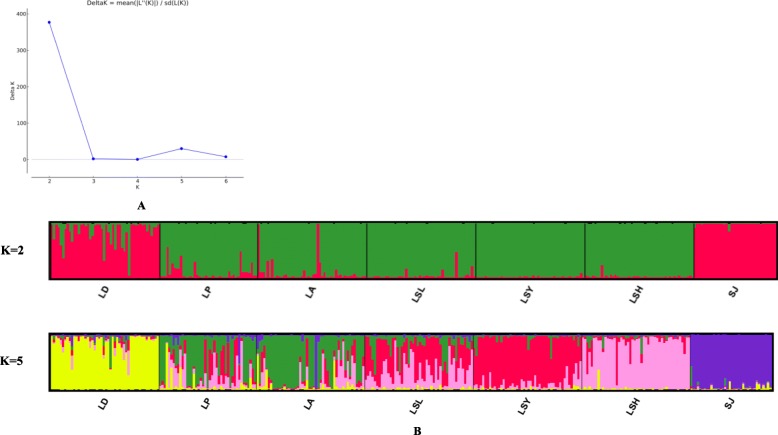
Bayesian clustering of all seven *P. sinensis* populations using 16 microsatellite markers. **a**: The ΔK values (K = 2–7). **b**: Analysis of seven populations (*n* = 319 individuals, K = 2 and 5)

The logarithm probabilities Ln P (X/K) related with different numbers of genetic clusters K, calculated from structure analysis of 319 individuals of *P. sinensis* showed the highest value at K = 2, and followed by K = 5. As shown in Fig. 3, based on the value of K = 2, the individuals from LD and SJ populations were merged to some extent and were obviously different from the individuals of the other five populations. Based on the value of K = 5, individuals from LD and SJ populations were significantly different from each other, and the other five populations were similar to some extent, with LP and LA as well as LSL, LSY and LSH being the more similar.

## Discussion

### Analysis of microsatellite polymorphisms

One of the main objectives of this study was to detect polymorphisms in transcriptome-derived microsatellite makers and to further understand the genetic diversity and structure of the wild populations from different locations. Compared with genomic derived microsatellites, transcriptome-derived microsatellites have several advantages including high efficiency, strong transferability, and correlation with potential genes [10]. Although transcriptome-derived microsatellites are predicted to be relatively less polymorphic than those derived from genomic DNA because they are more likely under stringent evolutionary constraint, some studies have given different results [17, 18]. In this study, 16 polymorphic microsatellites were identified, with the *N*_*A*_ value ranging from 2 to 13 (mean = 5.8), which is similar with studies investigating transcriptome-derived microsatellite markers in other decapod [10]. Additionally, compared to those genomic derived microsatellites in related species, such as *M. rosenbergii* [7] and *M. nipponense* [8], these 16 microsatellites present lower polymorphism. Since there is no report about genetic markers for *P. sinensis* so far, these polymorphic microsatellite makers and further mining of transcriptomic data may helpful for future research of *P. sinensis* and its related species.

### Genetic diversity within populations

The mean *N*_*E*_, *H*_*o*_ and *H*_*e*_ values in seven *P. sinensis* populations ranged from 1.441–1.867, 0.158–0.369 and 0.226–0.379, respectively, showing that the genetic diversity of the populations was lower than that of other species of the Palaemonidae family [7, 8]. Five populations including LD, LP, LA, LSL and SJ showed deficiencies of heterozygotes as indicated by significant *F*_*IS*_ values. The LD and SJ populations showed lower genetic diversity as indicated by *N*_*E*_, *H*_*o*_ and *H*_*e*_ values, which may be a result of a small *N*_*e*_ value, inbreeding and a limited number of shrimp samples. Results in the current study indicated that the *N*_*e*_ values of the four populations LD, LA, LSY and SJ, were lower than the values suggested by Franklin et al. [19]. This may due to the poor swimming ability of *P. sinensis*. In addition, due to time and space constraints in sampling, a certain number of individuals may have been from a few number of spawning shrimps, which would affect genetic diversity through an increase in genetic identity [20].

Most of the populations analyzed in this study showed a departure from HWE. This result was similar to several other studies which microsatellites derived from transcribed sequence data significantly depart from HWE [21–23]. This could be due to selection on polymorphisms in untranslated gene regions where these microsatellites typically reside, or to non-neutral dynamics of the genes to which they are physically linked [23]. However, the bottleneck effect and the M ratios indicated that all seven *P. sinensis* populations did not experience a bottleneck effect or a recent decline in quantity. Therefore, in future breeding projects, the level of inbreeding, brood shrimp population size and genetic diversity must be considered since domestication probably leads to a reduction in genetic variation due to genetic drift, selection and inbreeding [24].

### Relationships among different populations

In this present study, subjects are collected from three geographically isolated drainage basins. LD population belongs to Related River Drainage in East Liaoning Peninsula; the SJ population belongs to Huaihe River Drainage in north China and the other five populations (LP, LA, LSL, LSY and LSH) come from Liaohe River Basin in central Liaoning Province. Specifically, LSL and LSY come from a closed lake and a semi-closed reservoir respectively; LA and LSH belong to inland tributaries; LP locates close to the mouth of Liaohe River.

AMOVA analysis revealed that the genetic differentiation among populations of *P. sinensis*, which accounted for 17.24% of the total genetic variation, was much lower than that within populations. Pairwise *D* and *F*_*ST*_ values were consistent with these results. According to Thorp [16], the pairwise *D* values between most of the populations of *P. sinensis* indicated that they were closely related populations. Likewise, excluding SJ and LD populations, the pairwise *F*_*ST*_ values among the other five populations were low to moderate [15]. The slight and moderate differences of pairwise *F*_*ST*_ values among five populations indicated they might share the same ancestors. Even so, the significant correlation between geographical and genetic distances as well as the gene flow values indicated that five populations from Liaohe River Basin were separate due to the habitat fragmentation. The divergence between SJ and the other six populations as well as the divergence between LD and the other five populations were due to long-term geographic separation. These outcomes were consistent with the Bayesian analysis in genetic structure simulations, which also revealed that the SJ and LD populations were much different from the other populations.

## Conclusions

In this study, 16 polymorphic transcriptome-derived microsatellites were screened and used to assess the genetic diversity and structure among wild *P. sinensis* populations in China. All the polymorphic microsatellite makers are believed useful for evaluating the extent of genetic diversity and population structure of *P. sinensis*. Compared to the other five populations, the LD and SJ populations exhibited lower genetic diversity due to the lower *N*_*E*_, *H*_*o*_ and *H*_*e*_. All populations showed normal L-shaped distributions and may not have experienced a bottleneck or recent reductions in effective size. The AMOVA analysis revealed that genetic variation among populations was 17.24% and much lower than that within populations. *D* and *F*_*ST*_ values between any two populations indicated that the LD and SJ populations differed from the other five populations. The UPGMA tree and the STRUCTURE analysis also supported the result. Therefore, they needed to be protected against further declines in genetic diversity. Among the seven populations, LP, LA, LSL, LSY and LSH populations were all from Liaohe River Drainage with a relatively high genetic diversity, and hence can be considered as hot spots for in-situ conservation of *P. sinensis* as well as sources of desirable alleles for breeding values. In future, further development of transcriptome-derived microsatellite markers is necessary for more detailed investigation on the genetic variation, genetic structure, and molecular markers-assisted selection (MAS) of *P. sinensis.* Overall, this study provided a theoretical basis for the protection, rational use and genetic breeding of germplasm resources.

## Methods

### Sampling and DNA extraction

Totaling 319 individual shrimps from seven wild populations of *P. sinensis* were collected in 2016 from Sha River in Liaoning Dalian (LD, *n* = 48), Shuangtaizi River in Liaoning Panjin (LP, *n* = 43), Yangliu River in Liaoning Anshan (LA, *n* = 48), Longwei Lake in Liaoning Shenyang (LSL, *n* = 48), Yangshi reservoir in Liaoning Shenyang (LSY, *n* = 48), Liao River in Liaoning Shenyang Huangjia (LSH, *n* = 48) and Dushan Lake in Shandong Jining (SJ, *n* = 36) (Fig. 1, Table 1). Genomic DNA was extracted from the muscles of each shrimp using a TIAnamp Marine Animals DNA Kit (TIANGEN) according to the manufacturer’s protocol.

### Microsatellite selection and genotyping

More than 50 microsatellite loci were selected from the data of *P. sinensis* transcriptome (GenBank No. SRR5759507) [5], and all new primers were designed using Primer Premier 3.0 (http://bioinfo.ut.ee/primer3-0.4.0/). Primers were examined using varying PCR conditions and PCR amplified products were evaluated by agarose gels. PCR reactions were performed by ABI 2720 thermocycler (Applied Biosystems, USA). DNA samples were subsequently amplified for the polymorphic loci in single PCR reactions. PCR reactions were 15 μL, containing 50-100 ng template, 10 × buffer 1.5 μL, Mg^2+^ (25 mmol·L^− 1^) 1.5 μL, dNTPs (each of 10 mmol·L^− 1^) 0.25 μL, each of forward primer and reverse primer (10 μmol·L^− 1^) 0.15 μL, *Taq* polymerase 1 U, and ddH_2_O. Denaturation for 3 min at 94 °C was followed by 35 cycles made up of 25 s at 94 °C, 25 s at the 54 °C and 60 s at 72 °C. The final step was a prolonged extension of 10 min at 72 °C, followed by 4 °C hold. All individual genotypes were scored after the PCR products were resolved on Applied Biosystems 3730XL Genetic Analyzer (Applied Biosystems, USA) and the product size was analyzed by GeneMarker version 2.2.0. (Applied Biosystems, USA).

### Statistical analysis

Sixteen polymorphic loci were used to detect genetic variation among *P. sinensis* populations (Table 2). The number of alleles (*N*_*A*_), the number of effective alleles (*N*_*E*_), observed heterozygosity (*H*_*o*_) and expected heterozygosity (*H*_*e*_) of each locus of each population was calculated using POPGENE 1.32 [25]. Null allele frequency (*r*) in each locus was estimated using software FreeNA [26] in which loci with estimated frequencies of null alleles above 0.2 were considered as potentially problematic for calculations. Meanwhile, the allele richness (*A*_*r*_) and Wright’s *F*_*IS*_ values with 95% confidence intervals were calculated according to Weir and Cockerham using FSTAT version 2.9.3.2 software [27]. Tests for Hardy-Weinberg equilibrium (HWE) and linkage disequilibrium (Ld) were conducted using Arlequin 3.5.2.2 [28] and a Markov chain of 1,000,000 and 100,000 Dememorisation Steps.

The estimates of effective population size (*N*_*e*_) for each population was calculated using the gametic disequilibrium method implemented in LDNe 1.31 [29] based on the lowest allele frequency of 0.02 and confidence intervals estimated with the parametric method (which were highly similar to those estimated by the jackknife method). The mean ratio of the number of alleles to the range in allele size (M ratio) was used to assess recent changes in *N*_*e*_ value using Arlequin 3.5.2.2 [28]. Recent bottlenecks were performed using Bottleneck version 1.2.02 [30], under a two-phase model (TPM) with 90% single-step mutation. These methods test for departures from mutation-drift equilibrium based on heterozygosity excess or deficiencies. A Wilcoxon signed-rank test was used to determine whether a statistically significant number of loci displayed heterozygote excess compared to expectations based on the observed number of alleles.

Pairwise Wrights fixation index (*F*_*ST*_) and analysis of molecular variances (AMOVAs) were calculated using Arlequin 3.5.2.2 [28]. Cavalli-Sforza and Edwards’ genetic distance (D) [14], computed using the INA correction method described in Chapuis and Estoup, was also calculated using FreeNA [26], and then constructed using the dendrogram with genetic distance based on UPGMA cluster through POPTREE2 [31]. The significance was tested based on 1000 bootstraps. A Mantel test was performed to estimate a correlation between the matrices of genetic and geographical distances using Arlequin 3.5.2.2 [28] (10,000 permutations).

Pairwise gene flow among all seven populations was calculated by using software Migrate-n 4.4.3 [32, 33] through likelihood approach. The program was run with the following setting, 10 long chains of 10^5^, burn-in 1000, heating scheme (ten temperatures: 1.00, 1.12, 1.29, 1.50, 1.80, 2.25, 3.00, 4.50, 9.00, 1,000,000).

Genetic structures among populations analysis was performed via STRUCTURE v2.3.3 [34] using Bayesian methods. Parameters settings were assumed by an admixture model, with a burn-in of 50,000, with 100,000 Markov chain-Monte Carlo (MCMC) repetitions and 10 iterations per K (K = 2–7). The ΔK value was calculated by online software STRUCTURE HARVESTER [35] based on the rate of change in the log probability of data between successive K [36]. Plots of the clustering results were obtained using DISTRUCT [37].

## Supplementary information

**Additional file 1: Table S1.** *P*-value for deviation from Hardy-Weinberg equilibrium for each locus in each population.

**Additional file 2: Table S2.** Comparative migration estimation among all pairs of *P. sinensis* populations for coalescent (MIGRATE) estimator along with 95% confidence intervals.

**Additional file 3: Table S3.** Cumulative gene flow for each population of *P. sinensis.*

## Data Availability

Sequence data from this article have been deposited with the GenBank Data Library under the accession numbers: SRR5759507.

## Abbreviations

AMOVA: The analysis of molecular variance
*A*_*r*_: Allele richness
*D*: Cavalli-Sforza and Edwards’ genetic distance
*F*_*IS*_: Inbreeding coefficient
*F*_*ST*_: Wrights fixation index
*H*_*e*_: Excepted heterozygosity
*H*_*o*_: Observed heterozygosity
HWE: Hardy-Weinberg equilibrium
Ld: Linkage disequilibrium
Ln P: Logarithm probabilities
M ratios: The mean ratio of the number of alleles to the range in allele size
MAS: Molecular markers-assisted selection
MCMC: Markov chain-Monte Carlo
*N*_*A*_: Number of alleles
*N*_*e*_: Estimates of effective population size
*N*_*E*_: Number of effective alleles
PCR: Polymerase chain reaction
r: Null allele frequency
TPM: The Two-Phase Model
UPGMA: Unweighted pair group method with arithmetic mean
