# Supplementary material for: Genetic diversity and structure of Chinese grass shrimp, Palaemonetes sinensis, inferred from transcriptome-derived microsatellite markers
Source: BMC Genet. 2019 Oct 11;20:75. doi: 10.1186/s12863-019-0779-z (PMC6787973; doi:10.1186/s12863-019-0779-z)
Supplement: Supplementary file 1 — Additional file 1: Table S1. P-value for deviation from Hardy-Weinberg equilibrium for each locus in each population. [file 12863_2019_779_MOESM1_ESM.docx]

**Table S1 *P*-value for deviation from Hardy-Weinberg equilibrium for each locus in each population.**

| Locus | Population | | | | | | |
| --- | --- | --- | --- | --- | --- | --- | --- |
|  | LD | LP | LA | LSL | LSY | LSH | SJ |
| c119_g1_i1 | - | 1.0000 | 1.0000 | 0.0077** | 0.2712 | 1.0000 | - |
| c251_g1_i1 | 0.0549 | 0.4361 | 0.0369* | 0.2084 | 0.7790 | 0.2446 | 0.0092** |
| c317_g1_i1 | 1.0000 | - | 1.0000 | 0.0066** | 0.3096 | 1.0000 | - |
| c341_g1_i1 | 0.2618 | 0.0058** | 0.0000** | 0.1603 | 1.0000 | 0.0906 | 0.0007** |
| c864_g1_i1 | 1.0000 | 1.0000 | 1.0000 | 1.0000 | 1.0000 | 0.0550 | 1.0000 |
| c1089_g1_i1 | 0.5403 | 0.0100** | 0.0457* | 0.0109* | 0.0104* | 0.1103 | 1.0000 |
| c870_g2_i1 | 0.7684 | - | - | - | - | - | 1.0000 |
| c679_g1_i1 | 1.0000 | 0.9650 | 0.0911 | 0.7684 | 0.6535 | 0.0049** | 0.6752 |
| c792_g1_i1 | 1.0000 | 0.0003** | 0.0000** | 0.1488 | 1.0000 | 0.0041** | 0.0016** |
| c102_g1_i1 | 0.4061 | 0.0002** | 0.0245* | 0.0961 | 0.0122* | 0.0415* | 0.0000** |
| c1157_g2_i1 | 0.0227* | 0.1086 | 0.0073** | 0.0003** | 1.0000 | 0.0004** | - |
| c1198_g1_i1 | 0.0000** | 0.0000** | 0.0000** | 0.0103* | 0.4147 | 0.1165 | 0.0020 |
| c1730_g1_i1 | 0.3766 | 0.2693 | 0.3501 | 0.0142* | 0.3220 | 0.0555 | 1.0000 |
| c1747_g1_i1 | 0.3951 | 0.1631 | 0.0000** | 0.1025 | 1.0000 | 0.0146* | 0.0130 |
| c2017_g1_i1 | 1.0000 | 1.0000 | 1.0000 | 0.7464 | 1.0000 | 1.0000 | - |
| c2591_g1_i1 | - | 1.0000 | - | - | - | - | - |

*** significant(*P* < 0.05); ** highly significant (*P* < 0.01)**
