# Supplementary material for: Genetic diversity and structure of Chinese grass shrimp, Palaemonetes sinensis, inferred from transcriptome-derived microsatellite markers
Source: BMC Genet. 2019 Oct 11;20:75. doi: 10.1186/s12863-019-0779-z (PMC6787973; doi:10.1186/s12863-019-0779-z)
Supplement: Supplementary file 2 — Additional file 2: Table S2. Comparative migration estimation among all pairs of P. sinensis populations for coalescent (MIGRATE) estimator along with 95% confidence intervals. [file 12863_2019_779_MOESM2_ESM.docx]

**Table S2 Comparative migration estimation among all pairs of *P. sinensis* populations for coalescent (MIGRATE) estimator along with 95% confidence intervals**

| Pairwise  populations | 95% confidence intervals | Mean | Pairwise populations | 95% confidence intervals | Mean |
| --- | --- | --- | --- | --- | --- |
| M_LP->LD_ | 0.340-0.953 | 0.692 | M_LD->LP_ | 0.607-1.393 | 1.124 |
| M_LA->LD_ | 0.247-0.860 | 0.608 | M_LD->LA_ | 0.793-1.507 | 1.081 |
| M_LSL->LD_ | 0.307-1.013 | 0.731 | M_LD->LSL_ | 0.800-1.393 | 0.836 |
| M_LSY->LD_ | 0.367-0.960 | 0.711 | M_LD->LSY_ | 0.800-1.467 | 1.090 |
| M_LSH->LD_ | 0.340-0.987 | 0.721 | M_LD->LSH_ | 0.613-1.413 | 1.290 |
| M_SJ->LD_ | 0.420-1.080 | 2.014 | M_LD->SJ_ | 0.693-1.540 | 1.134 |
| M_LA->LP_ | 0.680-1.540 | 1.215 | M_LP->LA_ | 0.747-1.600 | 1.251 |
| M_LSL->LP_ | 0.493-1.180 | 1.896 | M_LP->LSL_ | 0.767-1.467 | 0.858 |
| M_LSY->LP_ | 0.673-1.507 | 1.263 | M_LP->LSY_ | 0.880-1.707 | 1.206 |
| M_LSH->LP_ | 0.613-1.400 | 1.212 | M_LP->LSH_ | 0.520-1.140 | 0.785 |
| M_SJ->LP_ | 2.473-4.880 | 3.545 | M_LP->SJ_ | 0.760-1.347 | 0.941 |
| M_LSL->LA_ | 0.640-1.460 | 1.219 | M_LA->LSL_ | 1.147-1.447 | 0.957 |
| M_LSY->LA_ | 0.653-1.293 | 0.962 | M_LA->LSY_ | 0.827-1.733 | 1.125 |
| M_LSH->LA_ | 0.607-1.413 | 1.099 | M_LA->LSH_ | 0.553-1.273 | 0.941 |
| M_SJ->LA_ | 0.687-1.420 | 2.400 | M_LA->SJ_ | 0.860-1.520 | 1.146 |
| M_LSY->LSL_ | 0.000-0.580 | 1.009 | M_LSL->LSY_ | 0.920-1.640 | 2.781 |
| M_LSH->LSL_ | 0.667-1.547 | 1.140 | M_LSL->LSH_ | 0.593-1.313 | 1.497 |
| M_SJ->LSL_ | 0.713-1.480 | 1.813 | M_LSL->SJ_ | 0.653-1.400 | 0.991 |
| M_LSH->LSY_ | 0.647-1.493 | 1.078 | M_LSY->LSH_ | 0.593-1.380 | 1.175 |
| M_SJ->LSY_ | 2.687-4.873 | 3.506 | M_LSY->SJ_ | 0.653-1.313 | 0.925 |
| M_SJ->LSH_ | 0.507-1.307 | 0.958 | M_LSH->SJ_ | 0.833-1.480 | 1.111 |
