# Supplementary material for: Genetic diversity and structure of Chinese grass shrimp, Palaemonetes sinensis, inferred from transcriptome-derived microsatellite markers
Source: BMC Genet. 2019 Oct 11;20:75. doi: 10.1186/s12863-019-0779-z (PMC6787973; doi:10.1186/s12863-019-0779-z)
Supplement: Supplementary file 3 — Additional file 3: Table S3. Cumulative gene flow for each population of P. sinensis. [file 12863_2019_779_MOESM3_ESM.docx]

**Table S3 Cumulative gene flow for each population of *P. sinensis***

|  | IMMIGRATION | EMIGRATION |
| --- | --- | --- |
| LD | 5.477 | 5.421 |
| LP | 10.255 | 5.733 |
| LA | 8.012 | 5.992 |
| LSL | 6.613 | 9.115 |
| LSY | 10.786 | 6.045 |
| LSH | 6.646 | 6.361 |
| SJ | 6.248 | 14.236 |
